# Anemia among children living with HIV/AIDS on HAART in Mekelle Hospital, Tigray regional state of northern ethiopia – a cross-sectional study

**DOI:** 10.1186/s12887-021-02960-1

**Published:** 2021-10-29

**Authors:** Feven Tesfay, Abrha Gebregerges, Haftay Gebrehiwot, Haftu Hailekiros, Letegebriel Girmay, Hadush Bekuretsion, Gebrekidan Gebrezigher, Gebreslassie Gebremariam, Gebreyohannes Teklehaimanot

**Affiliations:** 1 Department of Medical Laboratory Science, College of Health Sciences, Mekelle University, Mekelle, Tigray, Ethiopia; 2 Tigray Health Research Institute, Mekelle, Tigray, Ethiopia; 3 Department of Biochemistry, College of Health Sciences, Mekelle University, Mekelle, Tigray, Ethiopia

**Keywords:** Anemia, Children, HIV/AIDS, HAART, Tigray, Ethiopia

## Abstract

**Background:**

Anemia is a common complication of HIV/AIDS in children. There is lack of evidence on anemia prevalence among children living with HIV/AIDS on highly active antiretroviral therapy (HAART) in Tigray regional state, which the current study aimed to generate.

**Methods:**

An institution-based cross-sectional study was conducted on 241 children living with HIV/AIDS on HAART attending the antiretroviral therapy (ART) clinic of Mekelle hospital from November 2018-January 2019. Socio-demographic data were collected using a structured pretested questionnaire. Participants’ hemoglobin level was utilized to determine the prevalence of anemia. WHO cut-off values for Hgb were used to categorise the severity of anemia. Microscopic examination was performed for morphological classification of anemia.

**Results:**

Among the participants, 7 % (*n* = 16) were anemic in this study. Of these, 56 %, 19 %, and 25 % had mild, moderate, and severe anemia, respectively. Morphologically, normocytic-normochromic anemia was found the most common type of anemia in this study.

**Conclusions:**

The prevalence of anemia among participants was low in this study. However, a considerable proportion of participants had severe anemia, requiring regular monitoring of anemia status in these patients for better clinical outcomes and quality of life improvements.

## Background

Anemia is one of the most common and widespread disorders worldwide, which causes various public health problems [1, 2]. According to WHO, about 1.7 billion people are affected by anemia, and the condition has the most dramatic health impact in children with an estimated global prevalence of 43 %; of this, majority of them live in resource constrained settings in Africa [3, 4]. In Ethiopia, according to a 2016 national survey conducted by the Ethiopian Public Health Institute, the prevalences of anemia adjusted for altitude among preschool children and school-age children were 34.4 % (1 in 3) and 25.8 (1 in 4), respectively, with 31.6 % and 2.9 % in preschool children and 24.5 % and 1.3 % of the school children had moderate and severe anemia, respectively [5]. As per the reports of 2016 Ethiopian demographic health surveys, 56.9 % of children under-5 countrywide and 46 % in the Tigray region suffered from anemia [6].

Anemia is a common hematological complication associated with HIV infection in children, with its rate increasing with the progression of the disease [4]. It is a negative predictor of survival in these patients, posing a substantial negative impact on patients’ health, ranging from quality of life decrement to disease progression and decreased survival [7–12]. Due to high rates of malnutrition and iron deficiency [13], anemia has a substantial impact on children living with HIV/AIDS in developing countries such as sub-Sharan Africa with a prevalence range of 50–90 % [14]. Numerous studies from developed countries suggest the positive impact of HAART in reducing the risk and morbidity of anemia in HIV-infected children and improving their quality of life [10, 15, 16]. While other similar studies report anemia as the commonly observed complication associated with HAART, mainly with Zidovudine-based HAART [14].

In Ethiopia, a recent systematic review and meta-analysis [17] reported 22.3 % of the overall prevalence of anemia in children living with HIV/AIDS on HAART. The study also indicated that treatment with HAART was associated with a significant decrease in anemia prevalence. However, the review could not include any evidence from the Tigray region, stating that there was no study examining the prevalence of anemia among these patients in the region. This, to our knowledge, is the first attempt to assess the prevalence of anemia in children living with HIV/AIDS on HAART in the Tigray regional state of northern Ethiopia. Estimating anemia prevalence in these patients can offer guidance for local ART clinic managers and service providers to plan appropriate intervention strategies for better anemia management. The study will also have an important implication for program planners and decision-makers at various stages of the HIV/AIDS care and support program, including the regional health bureau. Further, the finding could be a good source of data for future systematic reviews in this space.

## Methods

### Study setting

 The study was conducted at Mekelle hospital, located in Mekelle, the capital of Tigray regional state, 783 km far north of Addis Ababa. Mekelle hospital serves as a referral hospital for the Tigray region, with a catchment population of about 8 million. It is the oldest general hospital in the region with a fully functional ART clinic and HIV/AIDS care services. The ART clinic has been serving the people living with the disease since 2003.

### Study design and period

An institution-based cross-sectional study was employed among children living with HIV/AIDS on HAART who had been attending the ART clinic from November 2018–January 2019. All children living with HIV/AIDS aged between 6 months to14 years on HAART whose parents/caregivers consented to participate were included in this study.

### Sample size determination and sampling procedure

A single population proportion formula was used to calculate the sample size. Using a 16.2 % prevalence of anemia in children living with HIV/AIDS on HAART in Northwest Ethiopia [18], a 5 % margin of error at a 95 % confidence level, and a 15 % non-response rate, the calculated sample size was 219. At the time of data collection, a total of 241 children living with HIV/AIDS were attending the clinic, and all the patients were therefore included in this study to provide a complete picture of anemia burden in the study population.

### Data collection and quality control

All study participants were approached during their respective appointments for a routine check-up and follow-up. Data on socio-demographic characteristics were collected by interviewing parents/caregivers using semi-structured and pretested questionnaires. The questionnaires were developed by reviewing the relevant literature and pretested in similar setups before the actual data collection to increase the quality of the data. The interview was performed after providing sufficient information about the study and securing informed verbal consent. The questionnaires were translated from English to the local language (Tigrigna) and back to English to maintain their consistency. Data were collected from those patients/caregivers by trained nurses and laboratory technicians working in the ART clinic. A one-day training was given on the overall interview procedure to minimize systematic errors and improve the validity of the data.

Laboratory investigation was performed by the same qualified laboratory technicians to measure the Hgb level of participants to determine prevalence of anemia. Laboratory test quality was assured by strictly following standard operating procedures (SOPs) for blood sample collection and analysis used for routine laboratory tests in the clinic. Accordingly, a blood sample (3-mL) was collected from each participant using vacutainer tubes containing 2.0 mg/mL ethylenediaminetetraacetic acid (EDTA-K2). The samples were processed and immediately analysed to avoid any impact from sample storage.

### Data analysis and interpretation

The filled questionnaires were checked for completeness and consistency before each study participant left the ART clinic. SPSS version 24.0 software was used to analyse the cleaned data. Frequency and percentage distribution were used to summarise and present the data. The collected samples were thoroughly mixed before analysis to avoid clumping and clotting of the samples. The Hgb value of each sample was then determined using a hematology auto analyser (SYSMEX-XT 4000i, Sysmex Corporation, Kobe, Japan) within 2 h of sample collection. The prevalence of anemia was determined according to WHO Hgb reference range [19]. The cut-off values included: <11 g/dl for children < 5 years old, < 11.5 g/dl for children 5–11.9 years old, and < 12 g/dl for children 12–14.9 years old after adjustment of altitude. The adjustment was done by subtracting 0.08 g/dl for an altitude of 2254 m in the study area [19]. Following this, the morphological characteristics of red blood cells (RBCs) of the anemic patients were visually examined using a microscope by qualified laboratory technologists and classified into various features based on the overall size of the RBCs.

## Results

### Socio-demographic characteristics

All (*n* = 241) the patients attending the ART clinic were interviewed, giving a response rate of 100 %. Table 1 presents the socio-demographic characteristics of the participants and caregivers. The mean age of the study participants was 9.3 ± 3.3 (range: 0.5–14) years. Half (51 %) of the participants were males, giving an almost 1to1 (male: female) ratio. Most of the participants were orthodox Christian. Only a few (12 %) of the patients’ guardians were educated, while about a quarter was unemployed.

**Table 1:** Socio-demographic characteristics of children living with HIV/AIDS on HAART at Mekelle hospital, 2019 (*n* = 241)

Variable	Frequency (n)	Percentage (%)
**Age (years) group**		
0.5–<5	22	9.2
5–<12	126	52.2
12–<15	93	38.6
**Gender**		
Male	124	51.4
Female	117	48.6
**Religion**		
Orthodox	221	91.7
Muslim	19	7.8
Protestant	1	0.5
**Educational status (Guardian)**		
Illiterate	25	10.4
Primary education	109	45.2
Secondary education	79	32.8
Tertiary education	28	11.6
**Employment status (Guardian)**		
Employed	58	24.0
Farmer	15	6.3
Daily labourer	72	29.9
Merchant	53	22.0
Unemployed	43	17.8

### Prevalence and type of anemia

Among the study participants, 7 % (*n* = 16) were anemic in this study. Among this, more than half (56 %, *n* = 9) of them had mild anemia, while a quarter (25 %, *n* = 4) of them were severely anemic.

**Fig. 1:**
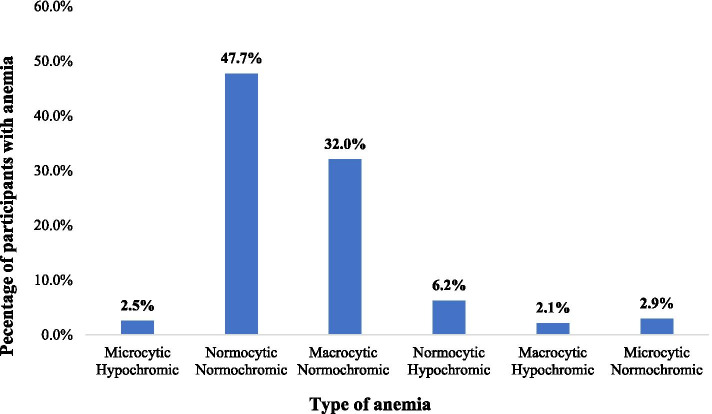
Morphology features of anemia in children living with HIV/AIDS on HAART at Mekelle hospital, 2019

On the morphology features of anemia, the majority (48 %, *n* = 8) of anemic children had normocytic-normochromic anemia followed by macrocytic-normochromic anemia (39 %, *n* = 6) (Fig. 1).

## Discussion

Despite several reports elsewhere in Ethiopia [17], this is the first study assessing the prevalence of anemia in children living with HIV/AIDS on HAART in the Tigray regional state. The prevalence of anemia in this study was 7 %, with most patients having mild to moderate anemia. This anemia prevalence is a mild public health significance as per the WHO classification [19]. This result is lower than the findings (11.4–39.4 %) reported in other similar studies in Ethiopia [17, 20, 21]. This variation could be attributed to the difference in socioeconomic status, geographical factors (altitude), nutritional factors, seasonality, WHO clinical staging of HIV disease, time of the study, or a combination of these factors, which are known to affect anemia [10–12, 17, 20]. It also seems clear from the results that HAART could be associated with the observed low prevalence of anemia in this study. Similar studies from Ethiopia also reported up to 60 % reduction in anemia prevalence in HIV-infected children on HAART compared with non-HAART initiated counterparts [17, 22, 23]. This is also in agreement with other studies elsewhere [10, 24]. This suggests that a sustainable effort would be imperative to further improving the quality of HAART service and ensuring optimum HAART adherence for better management of any health compromise from anemia.

Although anemia was less prevalent in this study, it is crucial to highlight the considerable proportion (25 %) of severely anemic participants. This result is higher than the findings reported in Northwest Ethiopia (2.3 %) [18], Southwest Ethiopia (14.3 %) [25], Southern Ethiopia (6.5 %) [21], Uganda (4.8 %) [26], and India (8 %) [27]. Given the high mortality rate of severely anemic children living with HIV/AIDS on HAART [28], this particular finding is a grave concern. Thus, we suggest taking this finding as a signal for routine monitoring of the patients’ hematologic parameters to ensure that morbidity is minimised and quality of life is optimised. It also is advisable to assess and manage any anemia predisposing factors as part of the routine monitoring. Additional specific treatments in this group of patients may be beneficial to correct the severe anemia.

On the type of anemia, normocytic-normochromic anemia appears to be more common (48 %) in this study. This finding agrees with studies done by Engdaw et al. (2015) [18] and Fenta et al. (2020) [21], which reported the occurrence of normocytic-normochromic anemia in 46.5 % and 64.5 % of participants, respectively. However a study conducted in Uganda reported microcytic-hypochromic anemia (44.9 %) as the predominant type of anemia [26]. Due to limited data, we could not suggest any underlying causes for the observed microcytosis and macrocytosis in the current study.

This study provides first-hand prevalence data on the study populations in our region. Interpretation of the findings should consider the following limitations, however. The findings may not be generalisable given the study population and anemic patients were relatively small to represent the whole children living with HIV/AIDS on HAART population in the region. In addition, the study did not explore the association between anemia and various factors such as socio-demographic characteristics, environmental, nutritional, disease-related, lifestyle, presence of malaria, and intestinal parasitosis, which may affect the Hgb levels of the study participants. Therefore, we were unable to identify the determinants of anemia in this study. Undertaking in-depth studies, exploring the underlying factors is deemed necessary.

## Conclusions

Among children living with HIV/AIDS on HAART in the Tigray region, the prevalence of anemia was low, with mostly mild in severity. Normocytic-normochromic anemia was the most frequent type. Although the overall anemia prevalence was relatively low, the presence of considerable severely anaemic patients suggests regular monitoring of anemia status for better clinical outcome and quality of life improvements. Further well-designed studies are required to evaluate the impact of HAART on anemia and survival of children living with HIV/AIDS on HAART at the regional level for conclusive evidence.

## Data Availability

All data generated or analysed during this study are included in this published article.

## Abbreviations

AIDS: acquired immunodeficiency syndrome
ART: antiretroviral therapy
HAART: highly active antiretroviral therapy
Hgb: hemoglobin
HIV: human immunodeficiency virus
